# Your brain on art, nature, and meditation: a pilot neuroimaging study

**DOI:** 10.3389/fnhum.2024.1440177

**Published:** 2025-01-20

**Authors:** Beatrix Krause-Sorio, Sergio Becerra, Prabha Siddarth, Stacey Simmons, Taylor Kuhn, Helen Lavretsky

**Affiliations:** ^1^Department of Psychiatry, Semel Institute for Neuroscience and Behavior, University of California Los Angeles, Los Angeles, CA, United States; ^2^Hope Therapy Center, Burbank, CA, United States

**Keywords:** brain, fMRI, art, nature, meditation, emotion

## Abstract

**Objectives:**

Exposure to art, nature, or meditation, all transcending human experiences, has beneficial effects on health and wellbeing. Focusing inward or watching art and nature videos elicits positive emotions that can help heal stress-related conditions. In a pilot functional magnetic resonance (fMRI) study, we explored the effect of watching digital art or nature videos compared to contemplating the universal connectedness (also known as transcendental meditation). The instructions were to meditate on the connection to a Universal Soul linked to a sense of expansion and universal connectedness (“one with everything”), which was prompted by a video of the galactic nebula that also controlled for the visual stimuli of the two other conditions.

**Methods:**

Nine healthy adults (mean age = 29; range = 19–42; 5 women) underwent a block design fMRI scan using a Siemens 3T Prisma scanner. The blocks included (1) nature videos, (2) AI-generated digital art (“machine hallucinations” by Refik Anadol), and (3) videos of NASA Webb-produced images of galactic nebulas. Brain oxygen-level dependent (BOLD) images were processed using FSL Version 6.0 and a general linear model (GLM) tested the contrasts between art, nature, and meditation blocks, using a cluster-corrected *p*-value of 0.05.

**Results:**

Compared to rest, meditation led to BOLD increases in bilateral lateral occipital and fusiform gyri, as well as right postcentral gyrus and hippocampus. Compared to viewing AI-generated digital art, increased BOLD responses during meditation were observed in left parietal and central operculum, and right pre- and postcentral gyri, and compared to nature, in the left parietal operculum, bilateral postcentral and supramarginal gyri, and bilateral lateral occipital cortices.

**Conclusion:**

Meditation compared to rest showed brain activation in regions associated with object, sensory, and memory processing. Meditation compared to nature videos led to activity in bilateral sensory and object processing areas, as well as a left sensory integration region (error monitoring), while meditation compared to art showed activity in left sensory integration and right sensorimotor regions. Further studies are needed to delineate the distinct neural signature and therapeutic effects of inner contemplation using human connection to art, nature, or meditative transcendent practices, in the brain and its potential in clinical applications.

## 1 Introduction

Transcendent experiences like being in nature, witnessing great art, or meditating on the universal connection to the Universe (e.g., transcendental meditation) can elicit a subjective sense of awe, joy and wellbeing, but can also change the perception of one’s life. There are numerous theories surrounding these effects. For example, biophilia theory suggests that humans have an innate attraction to nature, and the more recent stress reduction theory (SRT) postulates that exposure to nature reduces the effects of stress, possibly through homeostatic mechanisms (Christensen and Gomila, 2018; Kellert and Wilson, 1995). Thus, exposure to natural environments, compared to more perceptually demanding urban environments, can promote recovery from stress, reduce negative affect, and improve attention (Ulrich et al., 1991; Gaekwad et al., 2022; Gamble et al., 2014). In addition, a recent meta-analysis found that subjective measures of restoration from stress (e.g., perceived stress, restorativeness, or affect) were strongly associated with objective measures (e.g., blood pressure and heart rate) (Bolouki, 2023). Based on a meta-analysis, exposure to green spaces has been associated with decreased salivary cortisol, heart rate, high-density lipoprotein cholesterol, risk of type II diabetes, all-cause and cardiovascular mortality, increased heart rate variability, self-reported good health, and reductions in the incidence of stroke, hypertension, dyslipidemia, asthma, and coronary heart disease (Twohig-Bennett and Jones, 2018). An epidemiological study has demonstrated that after the COVID-19 pandemic, during which many became unemployed, worked from home during the lockdown, and experienced heightened stress, people have developed an even greater affection for green spaces and up to 4 times more people seek out conservation areas compared to before the pandemic, irrespective of the season (Tansil et al., 2022). Overall, nature exposure during the pandemic appeared to reduce depression, anxiety, and stress, and increased happiness and life satisfaction, whereby more nature exposure was associated with less physical inactivity and fewer sleep disturbances (Labib et al., 2022). Some have even argued that nature can induce intense emotions, including awe and inspiring energy, experiences related to spiritual transcendence (Bethelmy and Corraliza, 2019).

Several studies have reported positive effects of nature exposure in clinical settings, which further supports the SRT. Two decades ago, in-patients who underwent cholecystectomy were found to recover faster and needed less medication when they had a window view overlooking a natural environment compared to patients whose windows faced a brick wall (Ulrich, 1984). Yet, for many years, the notion that nature could facilitate recovery from a physical or mental illness was dismissed. However, more recently, there has been a resurgence of interest in this concept. A survey study found that women experienced greater pain relief from C-sections if they had a more subjectively satisfying window view (Wang et al., 2019). Similarly, having a picture of a natural scenery next to the bed during a painful bronchoscopy procedure reduced patients’ subjective pain experience significantly (Diette et al., 2003). Another meta-analysis found that interior design features in the hospital, including nature images, bigger rooms, and more sunlight had positive effects on anxiety and postoperative pain (Vetter et al., 2015). Thus, there is increasing evidence that not only being outdoors, but also exposure to more daylight and natural environments through a window or from pictures of nature has positive effects on stress and recovery from physical or mental illness.

Similar to effects from direct and indirect exposure to nature, production and passive experience of subjectively beautiful art have broad positive effects on wellbeing and have been widely applied in clinical settings. For example, art therapy can reduce anxiety, depression, fatigue, and quality of life in cancer patients, and depression in patients with neurocognitive and psychiatric disorders (Jiang et al., 2020; Batubara et al., 2023; Jenabi et al., 2023). While reduction of depression symptoms is frequently the therapeutic target, art therapy can also improve neurocognitive symptoms directly. For instance, the addition of virtual art therapy to physical therapy alone can improve independence in daily life activities and finger pinching strength in stroke patients (De Giorgi et al., 2023). The World Health Organization (WHO) identified over 3,000 studies on the effect of art on health and wellbeing and determined that art had positive effects on disease prevention, health, and the management and treatment of illnesses across the lifespan (Fancourt and Finn, 2019). In addition, the WHO concluded that early neuroaesthetics (appreciation of aesthetics in the brain) studies demonstrated reduced activity in the right caudate nucleus when viewing less preferred art pieces, and increased activity in bilateral occipital gyri, left cingulate sulcus, and bilateral fusiform gyri with greater preference for art pieces (Vartanian and Goel, 2004).

The WHO has argued that while being equivalent or even more cost-effective than common health interventions, art therapy targets a multitude of health outcomes and can be tailored to people of all cultural backgrounds, allowing for the inclusion of minority groups and populations that have limited access to standard medical treatment (Fancourt and Finn, 2019). While neuroaesthetics and art therapy are different disciplines, combining these could become a highly accessible and customizable form of adjunct therapy.

While producing art is not accessible to all, passively viewing art (also called “contemplation”), such as paintings or sculptures, requires fewer resources and has beneficial effects on health and wellbeing (Fancourt and Finn, 2019). Preliminary results demonstrate that viewing art images on a tablet might have beneficial effects on cognition, behavior, social relationships, and mood in older adults with dementia and their caregivers (Tyack et al., 2017). The same study also found that longer exposure durations led to greater improvements in these domains.

Due to its striking effect on human wellbeing and health, latest advances in neuroaesthetics explore the biopsychological mechanisms of art. The biopsychosocial and wellbeing effects that have been proposed to contribute to the observed cognitive and emotional effects of art include: (1) attentional focus and flow, (2) affective experience and higher affective sensitivity, (3) emotion elicited through imagery, (4) interpersonal communication, (5) self-intimation (observing one’s affective state introspectively), and (6) social bonding (Christensen and Gomila, 2018). These aspects have also been linked to subjective experiences of transcendence, which can be a spiritual construct for some and a construct of authenticity for others (Levin and Steele, 2005). For example, an association between the aesthetic experience of art and the ability to connect with spiritual or transcendent experiences has been reported (Świątek et al., 2023). Kawabata and Zeki (2004) found motor, parietal, lateral occipital, and orbitofrontal brain activity when viewing art. This suggests that there is a complex brain network involved in processing art, even when it is represented in pictures.

In a positron emission tomography (PET) study, participants watched film clips that evoked emotions and neutral clips, and were asked to connect with (“feel”) strong emotions they have recently experienced based on the presentation of prompted scripts (Reiman et al., 1997). The resulting film- and recall-generated emotion both led to significant symmetrical, bilateral increases in brain activity in the medial prefrontal cortex and thalamus. The authors argued that activity in these regions was specific to the internally generated emotional component, while increased activity in other regions reflected processing of other emotional components that were linked to external sensory features.

As discussed above, images have a profound impact on health, wellbeing, and neurophysiology. This effect appears to be modulated by subjective perceptions of aesthetics and the emotions elicited during image viewing. Positive emotions play a critical role in personal development, wellbeing, and social connection, and targeted therapy can enhance them (Helion et al., 2019; Fredrickson, 2001; Iwakabe et al., 2023). Emotion regulation is the ability to manage emotions both spontaneously and intentionally, and individuals possess varying levels of emotion regulation skills (McRae and Gross, 2020). This process involves adjusting emotions, including observing internal states, and is vital for psychological resilience, leading to improved coping mechanisms, health, and positive health behaviors (Cardi et al., 2021; Low et al., 2021; Rodriguez and Kross, 2023). Developing emotion regulation through therapeutic interventions that are calming or rebalancing can enhance mental and physical wellbeing.

Meditation is a common technique to disrupt ruminating thoughts and mitigate negative emotions, yielding benefits for psychological and physical health (Orme-Johnson and Barnes, 2014; Schneider et al., 2022; Hilton et al., 2017; Rogerson et al., 2024; Khanal et al., 2024). Transcendental meditation, a deep concentration on, and subjective connectedness with the cosmic consciousness, promotes a state of relaxed awareness (Travis, 2014). This practice fosters inner self-awareness, diminishes perception of time, space, and the body, and leads to higher brain integration, improved mood, emotional stability, and reduced anxiety (Orme-Johnson and Barnes, 2014; Travis, 2014; Mahone et al., 2018; Basso et al., 2019). Different meditation techniques target various cognitive tasks, from mindfulness and visualization to loving-kindness meditation and focusing on external stimuli like a candle flame. Utilizing transcendental meditation to expose novice transcendence practitioners to aesthetically pleasing images and guide them to connect subjectively with higher entities can enhance the therapeutic effects. Comparing the impact of transcendental meditation on anxiety and brain activity with exposure to art and nature in novice practitioners can offer valuable insights into its therapeutic mechanisms.

While nature, art, and meditative or contemplative practices engage distinct perceptual and cognitive processes, they all have the potential to reduce negative emotions, enhance psychological wellbeing, and facilitate transcendent experiences. Exploring the neural processing differences among these accessible activities in healthy adults, who are inexperienced in transcendental meditation can inform future therapeutic approaches for clinical populations. In the current pilot study in healthy young adults with sub-clinical levels of stress, anxiety, and depression, we used a whole-brain voxel-wise functional imaging approach to identify the brain regions that are specifically engaged when participants viewed natural scenes compared to artificial intelligence (AI)-generated digital art inspired by nature (“machine hallucinations”). Our hypothesis was that these stimuli would elicit activation in brain regions linked to visual processing, emotions, interoception, and memory. Additionally, we investigated brain activity in response to contemplating connection to the cosmic universal consciousness (transcendental meditation or visual stimulus-based inner contemplation), while participants viewed an aesthetically pleasing time-lapse video of night skies. We compared this brain response to that elicited when participants viewed videos of natural scenes and AI-generated art. We anticipated observing different individual neural signatures between all 3 conditions that can shed light on the neural mechanisms of transcendence.

## 2 Methods

### 2.1 Participants

Participants were recruited using study advertisements on websites, clinical trial registration, local advertisement posts, flyers spread across campus, the medical center, and neighboring communities. Inclusion criteria consisted of healthy, right-handed adults between 18 and 55 years old and without self-reported anxiety, depression, or chronic stress, which was later confirmed by baseline standardized questionnaires (see Table 1). Furthermore, participants were required to speak English fluently in order to comprehend the study instructions and materials, a Perceived Stress Scale (PSS) score greater than 1 and sufficient visual abilities to complete the assessments. Exclusion criteria included visual impairments that interfere with viewing of images on the screen, a history of psychiatric disorders, such as bipolar disorder, psychosis or intellectual disability, as well as neurological damage or impairment, alcohol, nicotine or substance abuse, suicidal attempts within the past 24 months, current use of antidepressant or anxiolytic medications. The following medical conditions were excluded: a history of serious, uncontrolled illness or medically unstable condition, including significant cardio-pulmonary disease, neurological disorders, seizures or epilepsy, cerebrovascular disease, thyroid dysfunction, diabetes. Contraindications for MRI included unsafe or unverified metal implants, pregnancy, claustrophobia, weight over 200 lbs. This study was approved by the University of California Institutional Review Board (IRB).

**Table 1:** Participant demographics and clinical scores.

Sample (N = 9)	Median	Range
Sex, *n* (%)		
Female	5 (55.6%)	
Male	4 (44.4%)	
Age	29	19–42
Education, years	16	13–18
BMI	22.4	20.6–36.0
PSS	22	15–27
PANAS		
Total	53	45–61
Positive affect	39	33–48
Negative affect	12	10–21
STAI-state (*N* = 9)		
Baseline	24	20–32
Post-scan	25	16–32
Signed rank test	*S* = −3, *p* = 0.73	

BMI, body mass index; N, total number of participants; n, number of participants; PSS, perceived stress scale; PANAS, positive and negative affect scale; STAI, state–trait anxiety inventory.

### 2.2 Task

During the scan, participants passively viewed 5 video blocks of nature scenes (e.g., national parks), 5 video blocks of AI-generated digital art (“machine hallucinations”), and 10 blocks of rest. Each block was 30 s long containing at least three different scenes each. Nature images were licensed from multiple commercial stock video libraries. Nature videos included images of waterfalls, flowers, snakes, forests, and shores (1 block each). The art blocks included morphing images of flowers, mountains and lakes, wave shapes, snakes, and waves consisting of morphing cubes. Art images were generated using artificial intelligence (AI) “machine hallucinations” and created by visual artist Refik Anadol. The task of contemplation on the universal connectedness (“meditation block” used for simplicity here) included 5 time lapse videos of the Milky Way and different cosmic nebulae. These aesthetic visual stimuli were intended to provide a similar task presentation mode across all 3 tasks. Participants were instructed to meditate on the Universal Soul and remember the expansive feeling of universal connectedness (“one with everything”) according to the training several days prior to the scan, while they were prompted by watching the images of cosmic nebulas.

### 2.3 Procedure

Participants were screened for MRI safety, demographics, health conditions, and eligibility over the phone. If participants were eligible, written informed consent was acquired at the in-person study visit. They were asked to abstain from caffeine, nicotine and alcohol at least 24 h prior to the scheduled visit in order to reduce external effects on the autonomic nervous system. Demographics [age, sex, number of years of school education starting in grade 1, body mass index (BMI)], neuropsychological questionnaires and neuropsychological tasks were acquired at the visit, as well as a one-hour MRI scan. Anxiety was assessed again after the MRI scan to test how the art/nature/meditation affected trait anxiety. Participants received instructions and practice for the meditation task prior to the MRI scan. If participants required vision correction, MRI-safe goggles at their required subscription were provided before the scan. Participants had the opportunity to select the lenses that were the most effective for them. Subsequently, participants underwent an MRI scan and completed the STAI-state a second time after completing the scan. All participants completed the task in the same scanner and under the same controlled conditions. The temperature was held constant between 68 and 72 degrees Fahrenheit (approximately 20–22 degrees Celsius), with the room lights turned off, and each participant had a light blanket on their legs.

### 2.4 Behavioral assessments

The Mini International Neuropsychiatric Interview (MINI) (Sheehan et al., 1998) is a brief structured diagnostic interview to screen for the 17 most common disorders in DSM-5. The Perceived Stress Scale (PSS) is a 10-item scale assessing subjective experiences of stress over the past month, whereby a greater number reflects higher levels of perceived stress (Cohen et al., 1983). The Positive and Negative Affect Schedule (PANAS) is a 20-item scale that consists of different words that describe feelings and emotions toward people and life experiences, each of which is rated using a five-point Likert scale (Watson et al., 1988). One subscale has positive and one has negative affect scales (10 questions each). The State–Trait Anxiety Inventory (STAI), a psychological inventory consisting of 40 self-report items on a 4-point Likert scale that assesses both state and trait anxiety (Spielberger et al., 1983). We assessed the 20 questions from the STAI-state portion.

### 2.5 FMRI tasks

In this block design, stimuli were back-projected onto a flatscreen located behind the scanner bore, viewed through an angled mirror mounted on top of the head coil. The resolution of the screen was 1,024 × 768 pixels, and the height of each stimulus was 18.85° (600 pixels), with varied width. Participants were presented with 10 blocks of images. Five blocks represented nature and five blocks art. Each block was based on a different rating category (beautiful, neutral, and unpleasant). Each block was presented for 120 s, with each image being displayed for 2 s. Eleven resting blocks were presented for 30 s and consisted of a centrally presented fixation cross participants were asked to focus on, while blinking normally, as needed. Participants were asked to press one of three buttons for each image, indicating whether it was beautiful, ugly, or neutral. A 120-s block at the end was reserved for the meditation task. The block duration of 120 s blocks was chosen based on a balance between prior meditation and video viewing and interoceptive state fMRI paradigms. Some visual stimulus-based meditation studies have used similar if not longer blocks to ensure participants viewed the presented videos long enough to provoke the desired effect [e.g., specific motional engagement (Davis Iv et al., 2008; Murphy et al., 2018; Brewer et al., 2011)]. On the other hand, some shorter block lengths ranging from 16 to 170 s have demonstrated robust responses to interoceptive engagement (Brefczynski-Lewis et al., 2007; Bauer et al., 2014; Weng et al., 2020; Baerentsen et al., 2010; Farb et al., 2013).

### 2.6 MRI acquisition

High-resolution T1-weighted and T2-weighted images and functional BOLD images were collected using a 3 Tesla Prisma-fit system (Siemens, Erlangen, Germany) and a 32-channel head coil. The multi-echo MPRAGE scan had the following parameters: 208 slices; isotropic voxels = 0.8-mm^3^; TR = 2,500 ms; TE 1 = 1.81 ms, TE 2 = 3.6 ms, TE 3 = 5.39 ms, TE 4 7.18 ms; TI = 1,000 ms; FoV read = 256 mm; flip angle = 8 degrees. Total acquisition time was 8:22 min.

Gradient-echo echo-planar imaging (EPI) scans were collected in the anterior–posterior direction with a total scan time of 28:27 min. Functional images involved the following parameters: 72 slices; isotropic voxel size = 2 mm^3^; multiband acceleration factor = 8; TR = 800 ms; TE = 37 ms; FoV read = 208 mm; flip angle = 52 degrees.

### 2.7 Analysis

#### 2.7.1 Data processing

Data were processed using FSL version 6.0 (FMRIB, Oxford, United Kingdom). Initial preprocessing steps included: motion correction (MCFLIRT) (Jenkinson et al., 2002) and 6 mm spatial smoothing. Motion artifact components were identified using independent component analysis-based automatic removal of motion artifacts (ICA-AROMA) (Pruim et al., 2015) and removed from further analysis. A 128-s high-pass temporal filter was applied to remove low-frequency drifts. Individual pre-processed functional images were co-registered to the respective structural images using FMRIB’s linear image registration tool (FLIRT) (Greve and Fischl, 2009). Subsequently, a non-linear transformation was applied using FNIRT (Anderson et al., 2010) to register the functional images into standardized Montreal Neurological Institute (MNI) space.

First-level analysis was performed with FEAT (FMRI Expert Analysis Tool) (Woolrich et al., 2004), including time-series statistical analysis using FILM with local autocorrelation correction (pre-whitening). Time series for each of the three conditions that were extracted (art, nature, and meditation) and registered into MNI space for group analysis with FLAME (FMRIB’s Local Analysis of Mixed Effects). Time series for each of the three task conditions were compared against the rest condition. Using a general linear model (GLM), the following contrasts were tested: art > nature, nature > art, art > meditation, meditation > art, nature > meditation, meditation > nature, as well as the average activity maps for art, nature, and meditation individually. Statistical significance was set at a two-sided *p* < 0.05. Lastly, cluster thresholding was performed using a Z-threshold of 3.1 (equivalent to an uncorrected voxel-level *p*-value = 0.001) and full connectivity matrix. The average signal of each resulting cluster was extracted for further correlation with the clinical scores. Temporal signal-to-noise (tSNR) maps of the whole-brain volume across participants were computed across tasks to demonstrate temporal stability of the task-related BOLD response relative to task-unrelated noise signals throughout the duration of the scan (Friedman and Glover, 2006). The tSNR was defined as the mean BOLD signal intensity of a voxel relative to the standard deviation of the signal over time.

### 2.8 Statistics

Using IBM SPSS Version 27, descriptive statistics were computed for demographics, psychological and neuropsychological scores. Pre- and post-MRI state anxiety (STAI-state) scores were compared using a non-parametric signed rank test with a two-sided alpha level of 0.05. The average cluster signals were each correlated with each clinical score using Pearson’s correlation coefficients and an alpha level of *p* = 0.05. Both uncorrected and Bonferroni-corrected are reported.

## 3 Results

Ten participants completed the baseline assessments. One participant was unable to complete the MRI scan. Participant demographics and scores are listed in Table 1. One of the nine participants was missing the meditation block and another participant was missing the post-scan STAI-state. There was no significant difference in state anxiety (STAI-state) between before and after the MRI scan (median change = −1, range = −5-7, *p* = 0.7). No other participants were excluded from the analysis. Temporal stability of the signal (a tSNR map) is depicted in Supplementary Figure S1.

**Figure 1:**
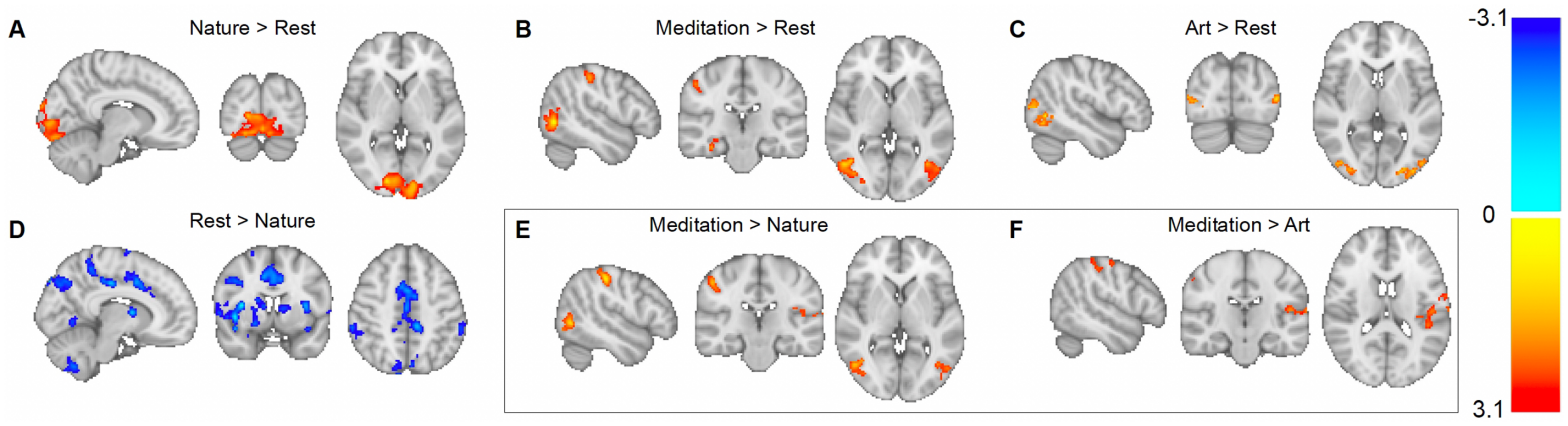
Brain oxygen-level dependent (BOLD) response to viewing nature, art, and meditation videos. **(A)** Compared to rest, nature led to a greater BOLD response across bilateral visual cortices; **(D)** while a reduced BOLD response was observed across a widespread network across both hemispheres; **(B)** compared to rest, meditation led to a greater BOLD response in bilateral lateral occipital cortices and fusiform gyri, the right postcentral gyrus and hippocampus; **(C)** compared to rest, art revealed clusters in bilateral lateral occipital cortices; **(E)** compared to nature, meditation showed greater BOLD responses in bilateral postcentral gyri, both the bilateral lateral occipital cortices, and the left parietal operculum; and **(F)** compared to art, meditation showed greater BOLD responses in the junction of the left central-to-parietal operculum and the junction of the right pre-to-postcentral cortex.

Cluster statistics are presented in Table 2 and depicted in Figure 1.

**Table 2:** Cluster parameters.

Hemisphere	Region	Cluster size (number of voxels)	*p*-value	Maximum intensity voxel	MNI coordinates		
					*X*	*Y*	*Z*
Meditation > Rest							
Left	Lateral Occipital Cortex	445	0.0000001	4.73	−40	−66	4
	Occipital Fusiform Gyrus	415	0.0000003	4.33	−18	−82	−16
Right	Lateral Occipital Cortex	569	<0.0000001	5.4	50	−66	0
	Occipital Fusiform Gyrus	410	0.0000004	4.07	18	−74	−10
	Postcentral Gyrus	218	0.000182	4.42	52	−26	48
	Hippocampus	92	0.0345	4.3	34	−24	−20
	Lateral Occipital Cortex, superior division	89	0.0399	4.12	30	−84	8
Art > Nature							
Left	Lateral Occipital Cortex	2,167	<0.0000001	7.05	−42	−74	−3
Right	Lateral Occipital Cortex	3,192	<0.0000001	6.91	44	−69	−3
Rest > Nature							
Left	Central opercular cortex/insular cortex	1,519	<0.0000001	8.18	−42	0	5
	Precuneus	820	<0.0000001	5.66	−12	−69	20
	Thalamus	292	0.00015	4.46	−5	−19	4
	VI (cerebellum)	273	0.00025	5.35	−30	−61	−28
	Crus II (cerebellum)	270	0.00027	4.8	−42	−54	−46
	VIIb	207	0.00172	4.41	−19	−71	−51
	Supramarginal gyrus	137	0.0167	4.16	−58	−38	44
	Frontal pole	123	0.0274	5.22	−38	50	18
	Basal ganglia	107	0.049	4.64	−10	4	7
Right	Central opercular cortex/insular cortex	2,793	<0.0000001	7.62	46	0	6
	Anterior cingulate	2,369	<0.0000001	6.09	1	−8	42
	Frontal pole	970	<0.0000001	6.25	36	49	17
	Precuneus	379	0.0000156	6.49	12	−76	39
	Lingual gyrus	369	0.00002	4.92	18	−57	1
	Basal ganglia	333	0.00005	5.7	18	2	7
	VIIIa (cerebellum)	210	0.00157	5.22	15	−66	−50
	Supramarginal gyrus	156	0.00875	4.58	56	−41	12
	Superior frontal gyrus	132	0.0199	5.01	17	−1	71
	Midfrontal gyrus/precentral gyrus	123	0.0274	4.45	42	7	35
Meditation > Art							
Left	Parietal operculum cortex/central operculum cortex	245	0.000118	4.16	−40	−68	6
Right	Postcentral gyrus/precentral gyrus	235	0.000166	4.28	−50	−30	56
Meditation > Nature							
Left	Parietal operculum cortex	358	0.000003	4.67	−60	−20	12
	Lateral occipital cortex	245	0.000118	4.16	−40	−68	6
	Postcentral gyrus/supramarginal gyrus	235	0.000166	4.28	−50	−30	56
Right	Postcentral gyrus/supramarginal gyrus	379	0.000002	4.75	52	−26	48
	Lateral occipital cortex	334	0.000007	4.48	50	−66	0

Compared to rest, the BOLD response during meditation increased in bilateral lateral occipital cortices, fusiform gyri, and postcentral gyri, as well as the right hippocampus. Compared to rest, nature led to a greater BOLD response across bilateral visual cortices. Compared to rest, nature led to reduced BOLD responses across a large network of left central opercular and insular cortices, precuneus, thalamus, cerebellar regions crus II and VIIb, supramarginal gyrus, frontal pole and the basal ganglia. In addition, clusters were found in the right central opercular and insular cortices, frontal pole, precuneus, lingual gyrus, basal ganglia, cerebellar region VIIIa, supramarginal gyrus, superior frontal gyrus, and midfrontal/precentral gyri.

Compared to rest, art revealed clusters in bilateral lateral occipital cortices. Two clusters were observed in the contrast meditation > art; the junction of the left central-to-parietal operculum and the junction of the right pre-to-postcentral cortex. Notably, no regions showed greater activation during the art block compared to the meditation block. The clusters resulting from the meditation > nature contrast overlapped with those observed in the meditation > rest average and included the bilateral postcentral gyri, both the bilateral lateral occipital cortices, and the left parietal operculum. Notably, there were no clusters for the contrast nature > meditation.

Pearson correlations between post-scan anxiety and cluster BOLD difference for the contrast meditation vs. rest were observed in the left occipital/fusiform cortex (*r* = −0.84, *p* = 0.01) and the right hippocampus (*r* = −0.74, *p* = 0.04; Supplementary Figure S2). These results did not survive correction for multiple comparisons (6 scores and 12 clusters = 72 tests; corrected *p*-value *p* = 0.000694). No further correlations were found (*p*’s > 0.36; Supplementary Figures S2, S3).

## 4 Discussion

In this cross-sectional pilot study in nine healthy, non-depressed adults, we found that in participants, who were first trained in transcendental meditation on cosmic Soul connection with the use of images of cosmic nebulas as a conditioned stimulus, and during an fMRI session were prompted to perform transcendental meditation practice with the visually-images of cosmic nebulas compared to rest, increased BOLD responses were observed in bilateral lateral occipital cortices, fusiform gyri, and postcentral gyri, as well as the right hippocampus. Art > rest engaged somewhat smaller and slightly disconnected regions in bilateral lateral occipital cortices. Compared to rest, nature videos elicited greater BOLD responses in bilateral lateral visual cortices and reduced BOLD responses in a widespread network across the brain, including left central opercular and insular cortices, precuneus, thalamus, cerebellar regions crus II and VIIb, supramarginal gyrus, frontal pole and the basal ganglia. In addition, clusters were found in the right central opercular and insular cortices, frontal pole, precuneus, lingual gyrus, basal ganglia, cerebellar region VIIIa, supramarginal gyrus, superior frontal gyrus, and midfrontal/precentral gyri.

During meditation compared to viewing art videos, increased BOLD responses were observed in the junctions between the left parietal and central opercula, and the right pre- and postcentral gyri. During meditation compared to nature, increased BOLD responses were observed in the junction between bilateral postcentral and supramarginal gyri, and bilateral lateral occipital cortices, as well as the left parietal operculum. Overlapping clusters were found in the right postcentral gyrus and the left parietal operculum for meditation > nature and meditation > art contrasts, while the left central operculum portion and the right precentral gyrus were unique to meditation > art, and bilateral lateral occipital cortices, right postcentral gyrus, and left supramarginal gyrus were unique to meditation > nature. Clusters in bilateral lateral occipital cortices were also found for the contrast meditation > rest and nature > rest. The lateral occipital cortex is part of the ventral visual stream, known for object recognition, symmetry, and size, and contributes to object completion by providing integrated feedback to lower visual regions, such as V1 and V2 (Zeng et al., 2020; Cattaneo et al., 2022; Chen et al., 2021). It has been found to code objects in a non-holistic format, independent of whether attention is employed (Guggenmos et al., 2015). Since the art videos in our current study included morphed pictures and not complete objects, it is likely that the lateral occipital cortex activity observed in the meditation condition and meditation > nature contrasts reflects the processing of visual object features.

Brain activity in pre- and postcentral cortices in the current study was observed in the right postcentral gyrus in the meditation condition, the right pre- and postcentral gyrus for meditation > art, and bilateral postcentral gyri for meditation > nature. This suggests the involvement of sensory and motor processing. Further evidence from functional and structural neuroimaging, circuit-based dissection, and human and animal behavioral studies suggests that motor and emotion networks are strongly interconnected, that emotion can modulate movement (Liang et al., 2016; Hassa et al., 2017; Braine and Georges, 2023) and that induction of negative emotion from pictures modulates human motor cortex plasticity and slows down motor speed in healthy adults (Li et al., 2019; Koganemaru et al., 2012). Such effects could result from the added cognitive resources required during an action task (Li et al., 2019). Further evidence from functional and structural neuroimaging, circuit-based dissection, and human and animal behavioral studies suggests that motor and emotion networks are strongly interconnected, that emotion can modulate movement (Liang et al., 2016; Hassa et al., 2017; Braine and Georges, 2023) and that induction of negative emotion from pictures modulates human motor cortex plasticity and slows down motor speed in healthy adults (Li et al., 2019; Koganemaru et al., 2012). It was suggested that such effects could result from the added cognitive resources required during an action task (Li et al., 2019). This is not only relevant for therapeutic interventions in movement disorders, but also for athletes and in professions that rely on the speed of their motor abilities, such as musicians, dancers, artists, and writers. It is noteworthy that bilateral somato-motor activity has also been observed in non-sensorimotor tasks, for example when individuals viewed art pictures they subjectively experienced as ugly compared to those they experienced as beautiful (Kawabata and Zeki, 2004). It is therefore possible that the observed sensorimotor activity in our current study was associated with subjective preferences of the presented pictures. However, due to the small sample size, we did not consider participants’ subjective ratings of beauty of the images.

Clusters in the parietal operculum were observed for meditation > art, as well as meditation > nature. This suggests that the meditation task involved brain processes unique to this task. Since the video images in the meditation task resembled aesthetic forms of a natural scene (i.e., had similar features to nature and art pictures), we believe the observed activity might stem from the cognitive or emotional functions that were engaged in the meditation task. The posterior operculum, a region adjacent to the insular cortex, has been associated with pain and temperature-related pain, tactile and vestibular functions (Garcia-Larrea et al., 2010; zu Eulenburg et al., 2013). The imagery in the meditation task was a time-lapse, locked-camera image of the night skies from the ground. This gave the illusion that the sky was rotating above the viewer. The posterior operculum may have been activated by this vestibular mimicry. It has also been associated with self-awareness during an error monitoring task (Terneusen et al., 2023). The authors argued that the posterior operculum is part of a fronto-parietal control network and demonstrated that low-performing traumatic brain injury (TBI) patients had greater activity in bilateral insular cortices and parietal opercula in TBI patients compared to control participants in response to errors. The effect was similar when comparing low-performing to high-performing patients, but with greater left lateralization. It is therefore possible that the operculum is involved in situations where the individual monitors internal states.

An increased BOLD response was also found in bilateral supramarginal gyri in the meditation > nature contrast. Greater activity in this region in individuals with anxiety and depression compared to healthy controls has been implicated in emotion and emotion regulation (Picó-Pérez et al., 2017). In addition, the left supramarginal gyrus has been associated with attention control to happy faces and the regulation of happiness in an emotional Stroop task (Loeffler et al., 2019). We therefore believe that this observed activity was associated with the emotion regulation component during the meditation task.

The clusters emerging from all three tasks compared to rest confirmed that participants experienced complex visual input. Additional clusters during meditation were found in the right hippocampus and postcentral cortex, which may be associated with memory retrieval of prior sensory experiences during prior meditation training.

There was a correlation between post-scan anxiety and meditation vs. rest cluster signal in the left occipital/fusiform cortex and the right hippocampus. However, these results did not survive correction for multiple comparisons. It is also noteworthy, that there was no correlation between cluster signal and baseline anxiety.

It is important to note that the cognitive and emotional aspects of art and nature exposure are complex and involve a variety of processes. For instance, both conditions in the current study involved a high degree of visual complexity, including light, shape, color, motion, composition, and places, and each individual may have experienced varying degrees of emotion, liking, and memory triggers for each picture (for a comprehensive review, see Cela-Conde and Ayala, 2018). For example, a relatively simple picture of a beach scene might trigger positive memories in some individuals, and no or even negative memories in others. Another complexity involves the temporal processing of pictures. In a magnetoencephalography (MEG) study it has been demonstrated that spatial and temporal components of art perception interacted with perceived beauty and non-beauty (Cela-Conde et al., 2013). Specifically, early signals for beautiful art pictures showed a different spatial distribution across the brain, compared to later signals. This pattern differed further for ugly pictures. The authors labeled these components the early and delayed aesthetic networks. This temporal component is important to consider because the relatively poor temporal resolution of the BOLD signal might not allow to control for the effects of what is perceived as beautiful or ugly. In the current study, images in the AI generated art were constantly morphing, video images in nature and the meditation were not static, and therefore, the temporal and motion components may have added additional noise to the effects. Other functions involved might be related to individuals’ body representation, which has been associated with the fusiform, precentral, postcentral, and supramarginal gyri, as well as the hippocampus, among other regions (Di Vita et al., 2016). The supramarginal, hippocampus, pre and postcentral gyrus are additionally associated with pain and pain-related conditioned fear (Biggs et al., 2020). It is possible that subjective feelings of emotional pain were triggered in the current study by any of the three task conditions.

There are several methodological considerations that may have influenced the outcomes of our study.

The primary limitation of this study was the small sample size, consisting of only nine participants. Consequently, we were unable to account for potential sex differences or consider participants’ previous experiences with art, nature, or meditation. In addition, we did not apply corrections for multiple comparisons between the 3 tasks. Despite the low statistical power, we were able to detect clusters consistent with the existing literature on watching videos of art and nature. For example, as discussed above, early viewing components differ from later ones (Cela-Conde et al., 2013), and people spend more time looking at imagery they find attractive compared to those they do not find attractive (Vartanian and Goel, 2004). The dynamic nature of our task may therefore have obscured results that could distinguish more carefully between preferred and non-preferred pictures. Overall, the BOLD signal does not allow for distinction of temporal components of this sensitive time frame, but adding additional task blocks for subjectively beautiful, neutral, and ugly pictures could remove the confounding effects of existing temporal dynamics. Future studies in larger samples will need to identify a network that can distinguish between art, nature, and meditation, with their transcendent nature, as well as considering esthetics of imaging.

Additional limitations of the study include the reliance on questionnaires and fMRI data obtained from a brief block design, which may limit the depth of data analysis. Furthermore, the art and nature blocks were each 120 s long. Given the extended duration of the entire scanning session, which exceeded an hour, it is uncertain if participants were able to fully engage with the images/videos throughout this period. It is also unclear to what extent fatigue effects may have impacted their responses and whether any lingering effects persisted into the subsequent 30-s rest periods. Furthermore, the fMRI design for the meditation task differed as it was presented in a single 120-s block instead of the block design used for art and nature stimuli. In future research, it may be beneficial to inquire with participants after each block regarding their level of engagement and connection to the stimuli, as well as whether the impact of the images persisted beyond the conclusion of each block. This feedback could provide valuable insights into how participants interacted with the stimuli and help interpret their neural responses more accurately.

Enhanced interpretability and broader applicability of the findings could be achieved by including assessments of visual acuity and color perception. Furthermore, using physiological measures like cortisol or heart rate variability instead of subjective stress reports may offer a more comprehensive understanding of stress responses. Future research endeavors could benefit from incorporating more extensive fMRI datasets, objective stress measurements, exploration of sex differences, and consideration of participants’ prior exposure to art, nature, and meditation. It is also recommended to conduct baseline resting state scans to examine the interplay between resting brain activity and baseline affect, perceived stress, and anxiety. These enhancements would provide a more holistic view of how viewing art, nature, and meditation videos impacts the brain networks associated with these psychological variables.

## 5 Conclusion

In conclusion, our pilot study demonstrates that there is an overlapping network of brain regions involved during watching art and nature videos. These regions include visual object processing, sensory, motor, and potentially self-monitoring and integration regions. Given that visual simulation and motion were shared features across all three tasks, the results likely reflect processes beyond simple visual stimulation. However, it is important to note that this pilot study is based on a small sample and therefore should be interpreted with caution. Future studies with greater statistical power should expand on the current results to identify more specialized networks for art, nature, and positive emotions by distinguishing the effects of participants’ subjective preference for pictures (beautiful, neutral, and ugly), and standardizing the visual features of the pictures (luminance, color, busyness or number of items or edges, and whether they evoke emotions or memories). Understanding brain networks underlying these features will support the refinement of clinical studies using pictures or videos as therapeutic interventions or help to develop cost-effective and low-tech clinical tools.

## Data Availability

The raw data supporting the conclusions of this article will be made available by the authors, without undue reservation.

## Supplementary material

SUPPLEMENTARY FIGURE S1 Temporal signal-to-noise (tSNR) map over the duration of the scan and across participants. Greater values (yellow) reflect greater task-related BOLD signal compared to noise.

SUPPLEMENTARY FIGURE S2 Uncorrected Pearson’s correlations between clinical scores (*x*-axis) and the cluster BOLD signal from the contrast meditation vs. rest (*y*-axis). The correlations between post-MRI state-trait anxiety and BOLD contrast difference in the left occipital cortex bordering on the fusiform cortex (*r* = −0.84, *p* = 0.01) and the right hippocampus (*r* = −0.74, *p* = 0.04) showed uncorrected *p*-values <0.05. Based on the number of correlation tests performed (72), these correlations would not survive correction for multiple comparisons (corrected threshold of *p* = 0.00069).

SUPPLEMENTARY FIGURE S3 Uncorrected Pearson’s correlations between clinical scores (*x*-axis) and the cluster BOLD signal from the contrast meditation vs. nature (*y*-axis). There were no significant correlations.
