# Supplementary figures and images for: Your brain on art, nature, and meditation: a pilot neuroimaging study
Source: Front Hum Neurosci. 2025 Jan 20;18:1440177. doi: 10.3389/fnhum.2024.1440177 (PMC11788305; doi:10.3389/fnhum.2024.1440177)

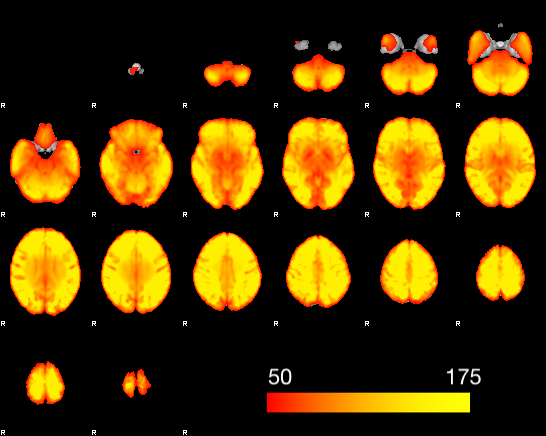

Supplement: SUPPLEMENTARY FIGURE S1 — Temporal signal-to-noise (tSNR) map over the duration of the scan and across participants. Greater values (yellow) reflect greater task-related BOLD signal compared to noise. [file Image_1.png]

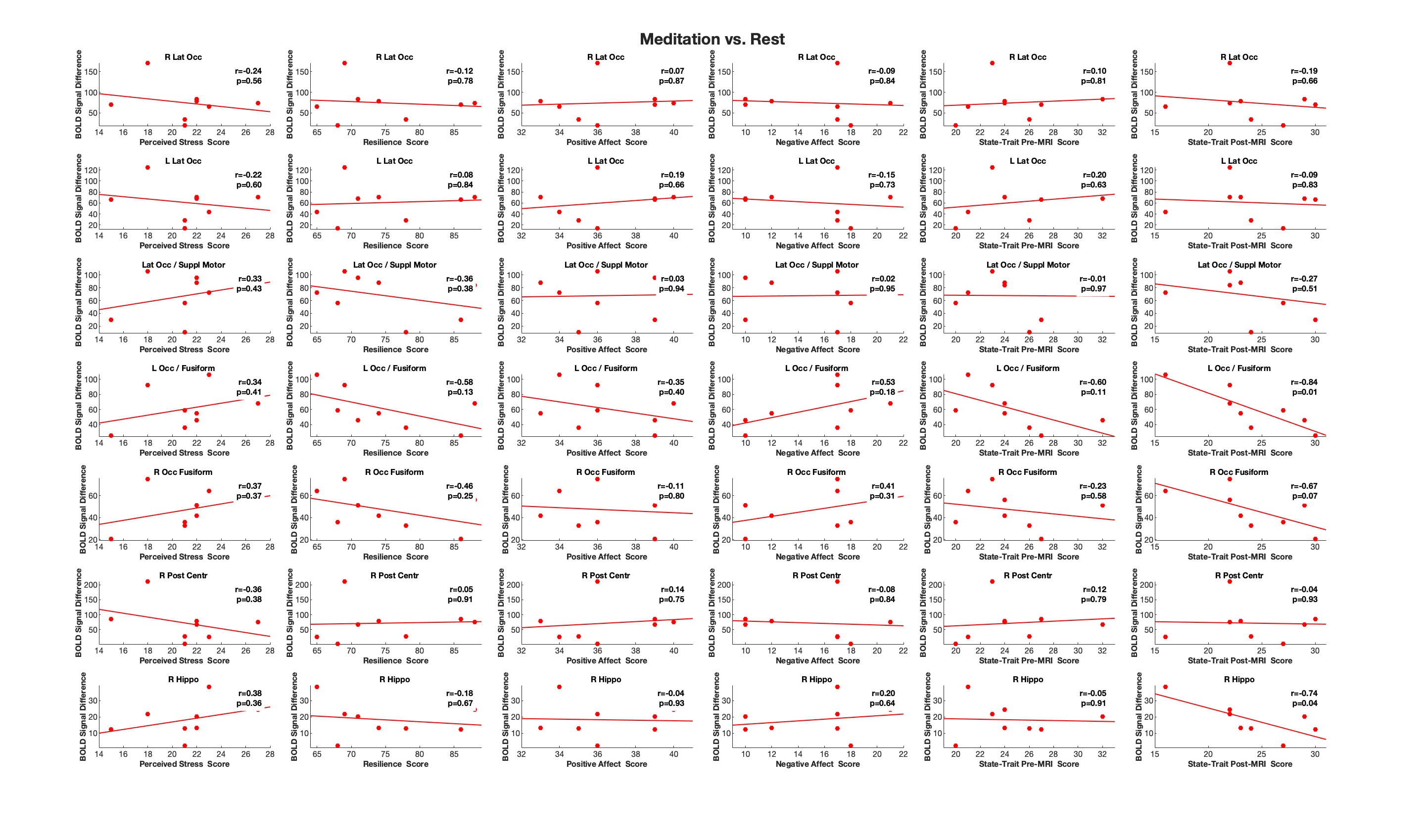

Supplement: SUPPLEMENTARY FIGURE S2 — Uncorrected Pearson’s correlations between clinical scores (x-axis) and the cluster BOLD signal from the contrast meditation vs. rest (y-axis). The correlations between post-MRI state-trait anxiety and BOLD contrast difference in the left occipital cortex bordering on the fusiform cortex (r = −0.84, p = 0.01) and the right hippocampus (r = −0.74, p = 0.04) showed uncorrected p-values <0.05. Based on the number of correlation tests performed (72), these correlations would not survive correction for multiple comparisons (corrected threshold of p = 0.00069). [file Image_2.jpeg]

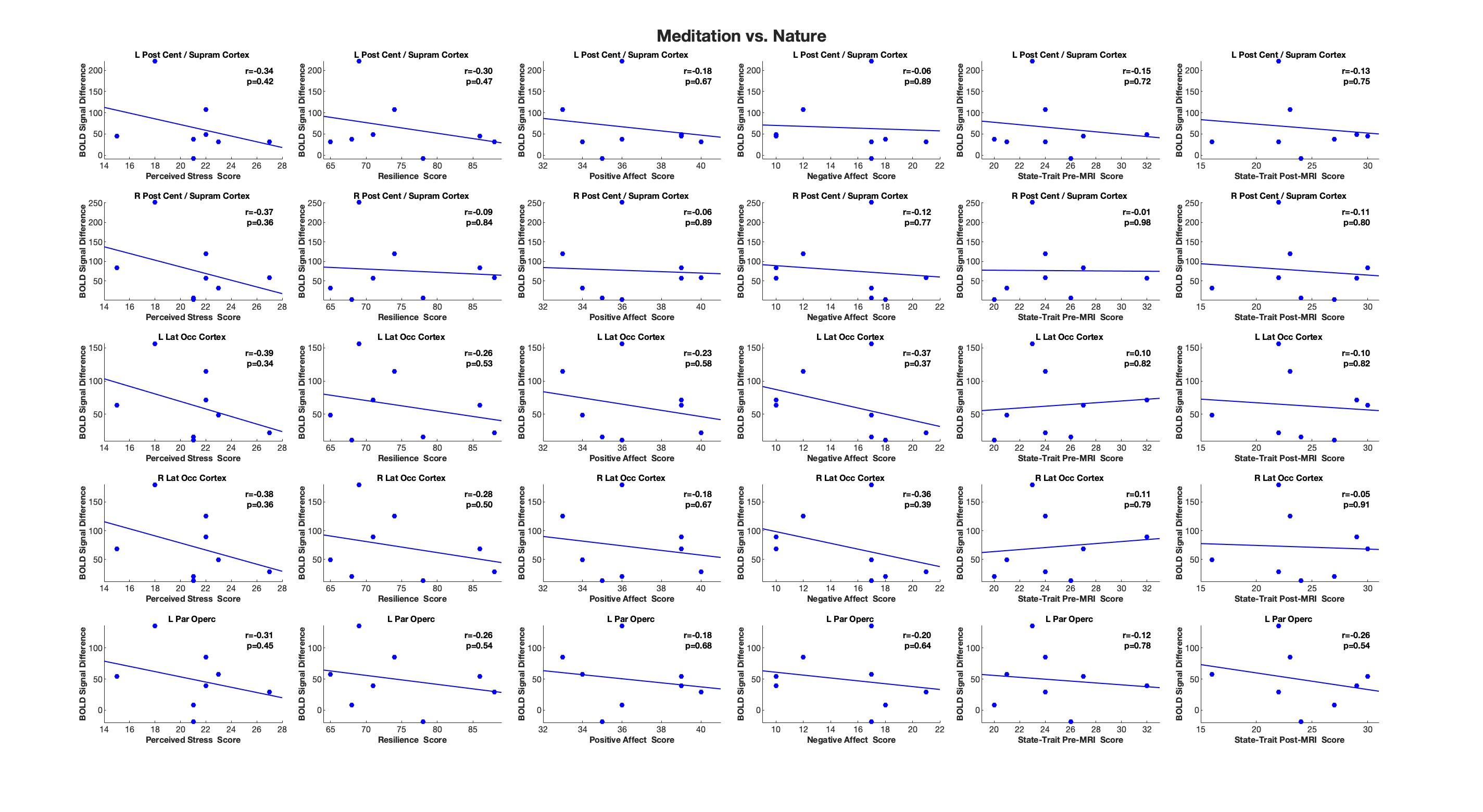

Supplement: SUPPLEMENTARY FIGURE S3 — Uncorrected Pearson’s correlations between clinical scores (x-axis) and the cluster BOLD signal from the contrast meditation vs. nature (y-axis). There were no significant correlations. [file Image_3.jpeg]
